# Fast Characterization of Surface Porosity in Additively Manufactured Components Using the Robust Analysis and Processing of Image Data (RAPID) Tool

**DOI:** 10.3390/ma19214527

**Published:** 2026-10-27

**Authors:** Mohanad Bahshwan

**Affiliations:** Department of Mechanical and Materials Engineering, Faculty of Engineering, University of Jeddah, Jeddah 21589, Saudi Arabia; mbahshwan@uj.edu.sa

**Keywords:** porosity, additive manufacturing, selective laser melting, pore characterization, pore morphology, RAPID

## Abstract

Porosity can significantly undermine the chemical and mechanical integrity of additively manufactured components. The porosity phenomenon is closely tied to the input variables, such as feedstock quality, environmental conditions, and 3D-printing operating parameters. A tool that can characterize porosity to establish useful correlations would aid in the optimization of the additive manufacturing process. However, a free open-source, intuitive, and workflow-capable tool for porosity characterization is not currently available. Here, a user-friendly, free, and open-source tool for robust analysis and processing of image data (RAPID) is introduced. RAPID was used in this paper to study the influence of varying laser powers on porosity count and morphology in additively manufactured 316L steel. It was found that lack-of-fusion pores diminished as laser power increased from 30 W to 70 W (75.8 J/mm^3^). Furthermore, increasing the power from 30 W to 90 W reduced the size of surface pores ten-fold. The data also suggested a 1.5-fold improvement in fatigue limit when power was increased from 30 W to 90 W. RAPID was validated side-by-side with a well-known image processing software, ImageJ, and was found to provide 18-times-more intuitiveness owing to a streamlined workflow. These results demonstrate some of the utility RAPID can introduce practitioners and researchers in materials science and engineering. RAPID is available for download from GitHub as an executable file on both Windows and Mac operating systems.

## 1. Introduction

Metal additive manufacturing technologies have proven useful in fabricating light-weight parts with complex geometries. One such technique is laser-powder-bed fusion (LPBF), which utilizes a laser beam to melt and subsequently fuse metal powder layer by layer. However, due to the nature of the constant melting, solidification, and remelting, some process-induced defects such as porosities can arise. These porosities can be detrimental to the integrity of LPBF components on several fronts. On the one hand, porosities can significantly impact the mechanical properties of LPBF components such as yield and fatigue strengths [1,2,3]. On the other hand, the pores often act as precursors to pit initiation in the corrosion of LPBF components [4,5]. Therefore, the additive manufacturing community continues to investigate and try to eliminate such phenomena.

**Figure 1:**
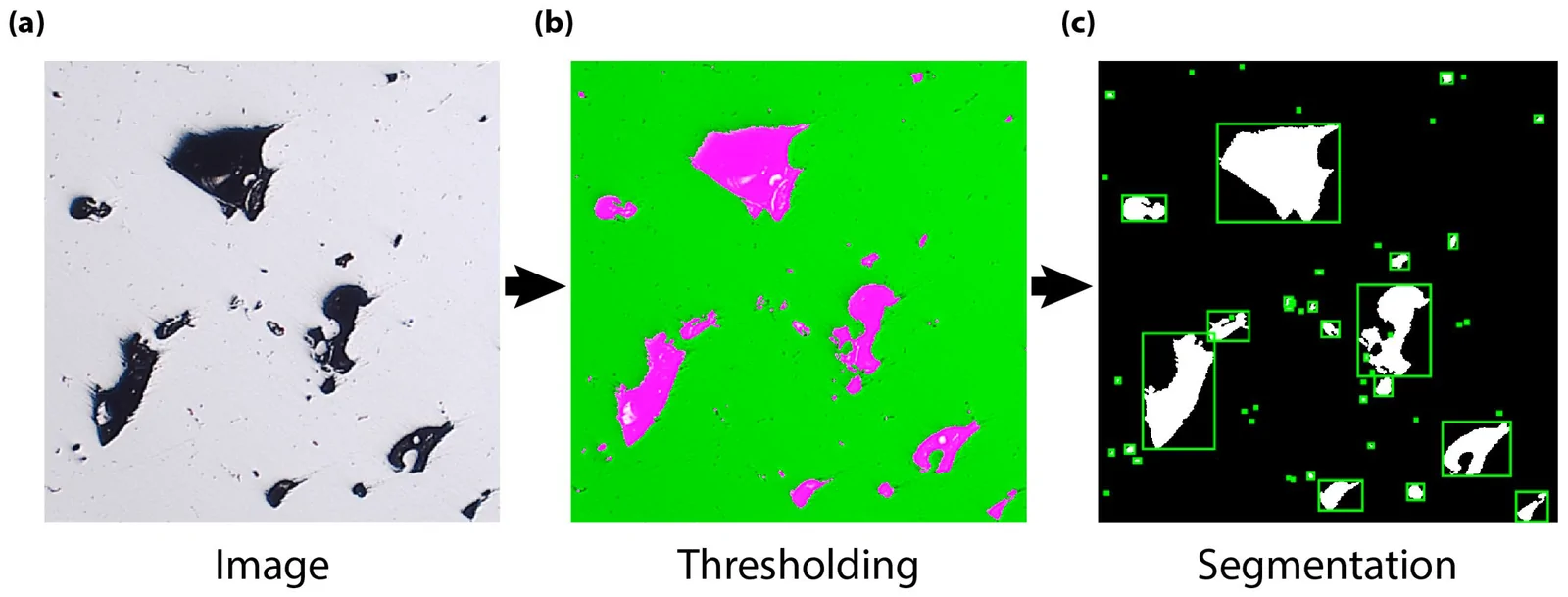
Standard workflow of segmentation technique via thresholding. The image shows porosities of additively manufactured 316L stainless steel.

There are several techniques for investigating porosity ranging from highly sophisticated to rudimentary ones. The X-ray computed tomography (CT) technique can provide precise quantitative and morphological characterization of porosity in three-dimensional form. However, the CT technology is the most expensive technique for porosity measurement [6]. On the other hand, the Archimedes method estimates part density from the mass of the specimen in air and in a fluid of known density. However, this method yields a single volume-averaged value without information on the size, shape, or distribution of the pores. An alternative approach relies on an optical digital microscope combined with image processing to quantify and characterize porosity of a surface [7,8]. In this approach, images are first captured with an optical microscope (Figure 1a). The images are subsequently processed by a thresholding technique (Figure 1b). Then finally, the objects (i.e., porosities) within the image are segmented and analyzed (Figure 1c). Despite it being a 2D technique, its low cost combined with its ability to provide morphological information makes it an attractive approach.

There are a number of options when users opt for image segmentation techniques. Some software is proprietary (e.g., Axiovision and Image-Pro) [9,10], whereas others are free (e.g., ImageJ/Fiji) [8,11,12,13]. While the proprietary software offers streamlined interfaces and features, they come at a prohibitive cost to many labs. On the other hand, the free tool ImageJ/Fiji has its own shortcomings. Given that ImageJ/Fiji is the de facto standard in biological research, its use outside life sciences is rather uncommon. ImageJ/Fiji’s complex interface and unintuitive design present a barrier to entry for non-computational life scientists who are consistently trained to use it [14]. For instance, ImageJ/Fiji relies on an unguided workflow where the user is presented with more than 160 options to select from upon opening the software. Such a decision-intensive step makes the software unintuitive. Intuition according to William James [15] can be loosely defined as the immediate, direct grasp of how things are connected or related, rather than a step-by-step reasoning about them. As a means for delivering an intuitive solution (i.e., enhancing user experience), Cooper [16] proposed to minimize unnecessary interactions with software. One of the key guidelines he proposed is to reduce the number of places a user can ‘go’ in the software. This may be done via guiding the user through a linear sequence of steps from start to finish. Another generally accepted guideline for delivering an intuitive software is to limit the software complexity to necessary parts [17]. While seemingly straightforward, adhering to these guidelines consistently presents an ongoing challenge for software development teams. The cognitive load theory proposed and developed by Sweller and Paas et al. [18,19,20] stipulates that meaningful learning can commence only after overcoming the cognitive overload of processing simultaneous information elements and interactions. In other words, a new user of a tool such as ImageJ/Fiji can only begin to learn it after processing the interface elements presented at each step and how they interact. It becomes apparent therefore why a new user may find ImageJ/Fiji unintuitive in their first interaction with it. Moreover, ImageJ/Fiji does not provide an easy way to develop an automated workflow of image segmentation for researchers to facilitate collaboration. According to Wang et al., such workflow for porosity investigation is lacking in current research [21]. As such, arbitrarily set parameters (e.g., morphological parameters, thresholding parameters, number of images acquired and processed) are difficult to track, making objective comparison between studies near impossible to perform [21].

It follows that there is a need for a tool that satisfies the following conditions: (1) free and open-source, (2) intuitive and user-friendly, and (3) capable of supporting a reproducible workflow. Hence, this study introduces robust analysis and processing of image data (RAPID) as a free and open-source, intuitive, and workflow-capable tool—satisfying all three conditions. This study will demonstrate the utility of RAPID for characterizing surface porosities in metals manufactured by LPBF. In particular, porosities in LPBF 316L stainless steel will be characterized as a function of laser power while keeping all other parameters constant. RAPID can be installed and used for free using the executable file on the GitHub repository: https://github.com/mohanad-bahshwan/RAPID (accessed on 8 October 2026). A video demo of RAPID’s use can be found in Supplementary Materials Video S1.

## 2. Materials and Methods

### 2.1. Sample Fabrication and Preparation

Disk-shaped specimens were prepared from 316L stainless steel gas-atomized powder (Table 1) using an LPBF printer Concept Mlab cusing (ConceptLaser, Lichtenfels, Germany). The machine uses a fiber-laser with a nominal beam diameter of 50 µm. The laser power was varied from 30 W to 90 W in steps of ten. The scan speed was held constant at 600 mm/s. The hatch spacing and layer thickness were 77 µm and 20 µm, respectively. The volumetric energy density (VED) for each type is shown in Table 2 for reference. Three replicates were printed of each specimen type. The scanning strategy was unidirectional with no rotation between layers. Samples were polished to a standardized roughness of R_a_ < 800 nm (≤N6 as per ISO 1302:1992 [22]). The polished surface corresponded to the scanning direction-transverse direction planes (i.e., looking into the build direction). Specimens were cleaned in an ultrasonic bath to remove any loose debris.

### 2.2. Image Capturing Protocol

**Figure 2:**
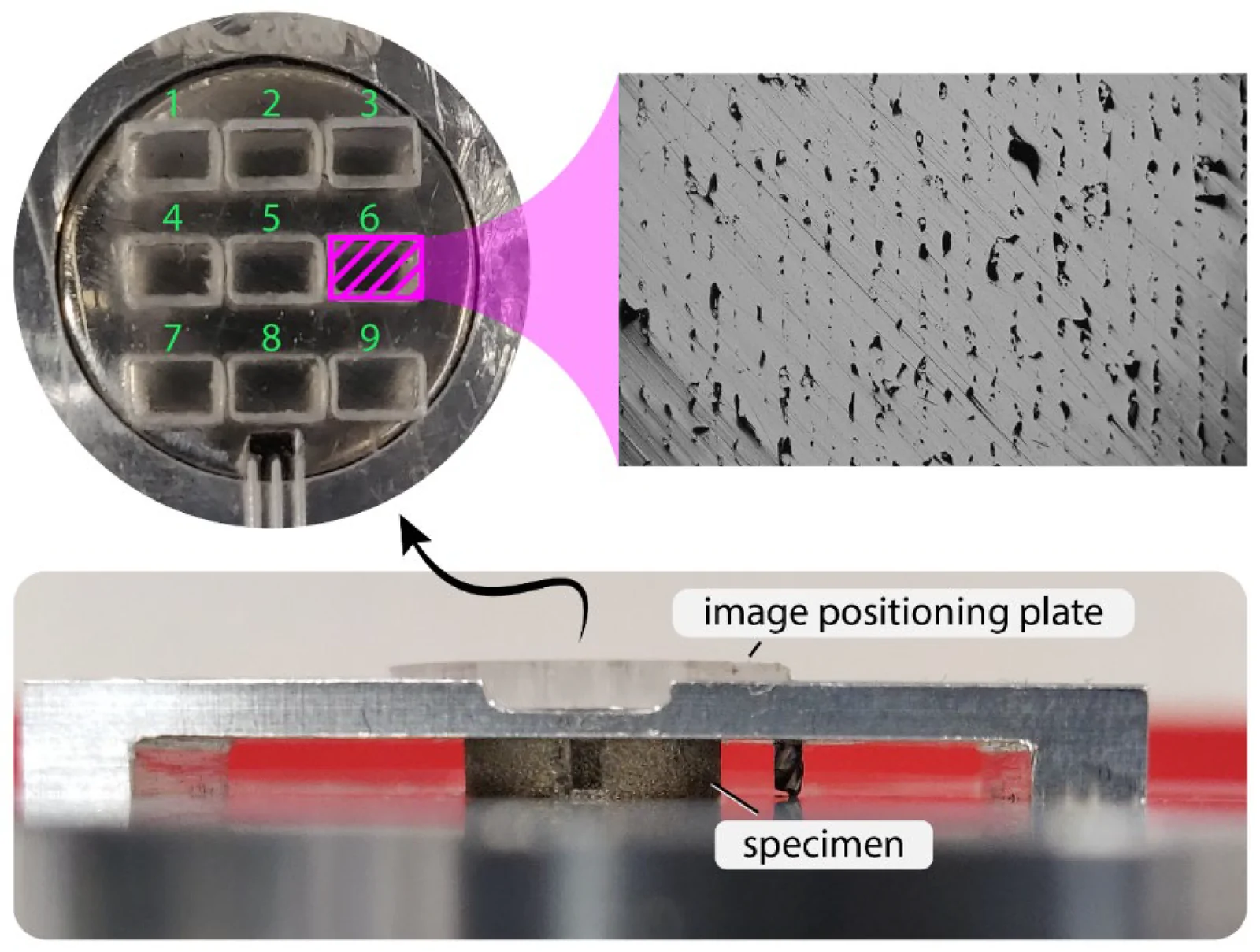
Imaging kit used to ensure consistency of microscope-imaging locations across different specimens.

Microscopic images were captured using a Hirox RH-2000 digital microscope (Hirox Co., Ltd., Tokyo, Japan) with 20× magnification lens in bright-field mode. To ensure consistency of imaging positions across samples, a custom kit was fabricated. The kit, shown in Figure 2, consisted of an aluminum specimen holder, an acrylic positioning plate, and an aluminum plate holder. The setup allows for image capturing without having to remove the acrylic positioning plate, thereby maintaining a consistent positioning across different specimens. The acrylic plate contained nine imaging positions as seen in the figure. For each specimen type and replicate, images were captured for all nine positions. Some artifacts (e.g., debris, scratches, shadows) were manually removed by a standard paint software before being processed into RAPID to ensure they are not counted as porosities.

### 2.3. RAPID Tool Design

**Figure 3:**
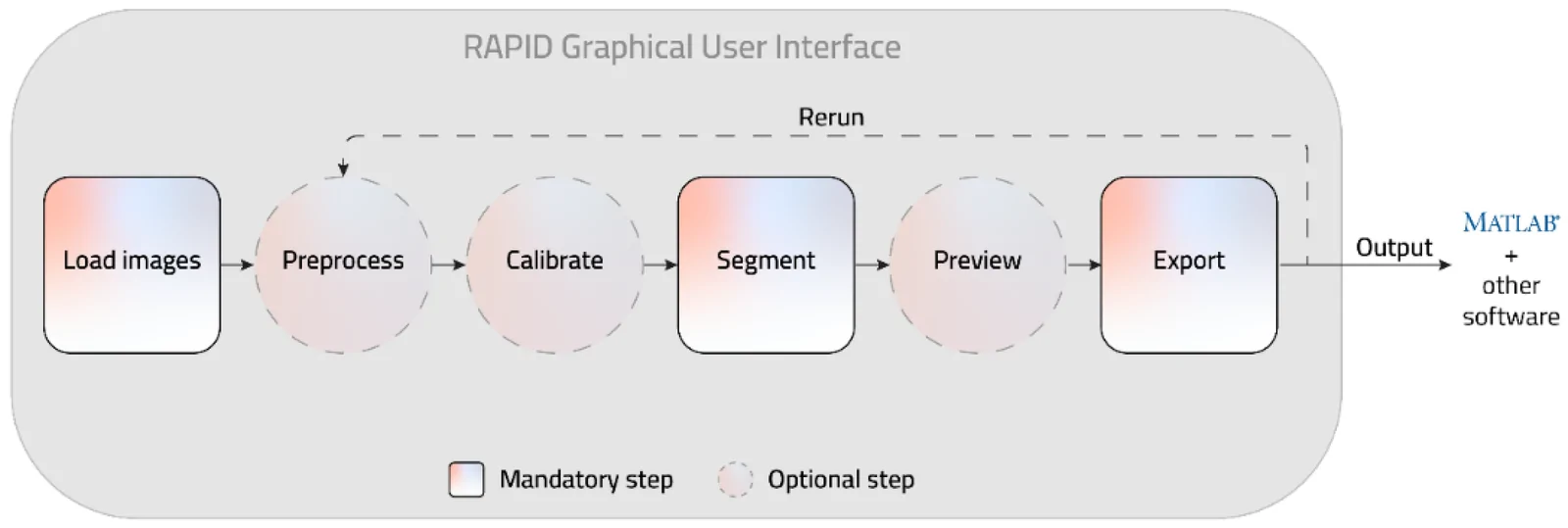
The stepwise workflow of RAPID showing mandatory and optional steps. Output can be processed by any comma-separated-values capable software. MATLAB^®^ can be used to expand the data processing capability (e.g., processing images, struct file). The logo of MATLAB^®^ was obtained with permission from MathWorks^®^.

The RAPID tool was completely written in MATLAB App Designer R2024b (The MathWorks Inc., Natick, MA, USA) and offered as a graphical user interface (GUI). RAPID was designed with a stepwise workflow to simplify the image segmentation process for the user (Figure 3). Upon loading RAPID, the user is guided through a sequence of mandatory and optional steps starting from loading the image all the way to receiving the export package. This workflow starts with a prompt to load the image(s), followed by two optional steps: pre-processing and calibration. The former of the optional steps allows the user to perform operations on the image(s) as needed such as cropping, enhancing contrast of edges, and removing microscope vignetting artifact. The latter allows the user to calibrate the units of measurements to a known scale, thereby converting from units of pixels to mm, μm, nm, etc., as needed. The next step is the actual segmentation step, in which the user selects the morphological operator and fine-tunes the segmentation parameters. Next, the user can choose to interact with the segmented objects in real time to analyze their morphological characteristics in the preview step. The final step in RAPID is the export package where the user specifies what they would like to include or exclude as part of their exported data. The dashed line in Figure 3 indicates that part of the exported package can be fed back into pre-processing. This feedback is done through a *preferences.m* file which may be reloaded and applied to a new dataset. This step is particularly useful for users processing multiple datasets that require identical image processing (i.e., workflow support). The user can then analyze the exported data on their preferred software such as MATLAB^®^ or another software of their choice. In mathematical terms, RAPID maps every imported image (*I_k_*) into a tabulated-statistics format (S*_k_*):

(1) $$S_{k}=K_{\kappa }\left(P\left(E^{\;e}\left(\Theta_{T}\left(\gamma_{B}\left(N^{\;n}\left(F^{\;\phi }\left(C_{R}^{\;\rho }\left(Y\left(U\left(I_{k}\right)\right)\right)\right)\right)\right)\right)\right)\right)\right),$$

where the subscript *k* denotes the image number *k* = 1,2,3 … *K*. U is the image-set harmonization function used to enforce all images to identical dimensions and channel-count. Y is the grayscale conversion function. $C_{R}$ is the crop to the region-of-interest function. *R* is the rectangle position and size. $C_{R}$ can be switched on or off with the crop Boolean flag *ρ* set to 1 or 0, respectively. F is the image uniformity correction (IUC), which removes vignetting effects. The IUC can be switched on or off with the IUC Boolean flag *ϕ* set to 1 or 0, respectively. N is the image inversion function which inverts the grayscale intensities. The superscript *n* can be set to 1 or 0 to enable or disable the image inversion, respectively. *γ_B_* is the grayscale morphological opening with the structuring element *B*. *B* can hold several structuring preset elements (disk, diamond, octagon, line, rectangle, square). *Θ_T_* is the global thresholding function at the threshold *T* (*T* ∈ [0, 1]). ℰ is the enhanced edge detection (EED) function which can be enabled or disabled with the EED flag *e* set to 1 or 0, respectively. P is a custom-written routine that distinguishes between border-touching objects and non-border-touching objects. It works by isolating the border-touching objects and subtracting those objects from the binary image to obtain the interior objects. This yields two images: one where all the objects are border objects and one that has no border objects. *regionprops()* is then applied to each image (refer to Mathworks [23] for more detail about *regionprops()*). For the first *regionprops()* table, a new Boolean field named BorderObject is added and set to 1, indicating that all those objects are border objects. For the second *regionprops()* table, the same Boolean field (*BorderObject*) is introduced and set to 0, indicating that all those objects are non-border-touching. The results are then concatenated into one monolithic table. K is the unit conversion function used to convert from pixels to the physical length unit selected by the user.

Further details and explanations of the features are available in the Supplementary Materials provided. RAPID is available for download from the official GitHub repository: https://github.com/mohanad-bahshwan/RAPID, (accessed on 8 October 2026).

### 2.4. Image Segmentation Parameters and Porosity Measurement

All images were processed in RAPID (MATLAB R2024b). The pixel size was calibrated in RAPID against a scale bar with known length which resulted in 1 pixel = 1.0777 µm. All images were loaded simultaneously, with each image converted to grayscale automatically. Because pores appear dark against the polished matrix, the images were inverted (in RAPID) so that pores became the bright foreground. The image uniformity correction (IUC) feature was applied to remove vignetting. Images were then processed by a grayscale morphological opening using a disk-shaped structuring element of radius 3 px. The morphological operator removes features narrower than the disk’s diameter (i.e., it sets the minimum detectable pore size). Images were then binarized with an intensity threshold of 0.587. The thresholding value was determined by using RAPID’s live overlay of the binary result on the grayscale image. This feature allows for visualizing the segmentation results before committing to the full computation, thereby saving computing time. Porosities intersecting the image border were included in determining the porosity % in the field of view. However, because these porosities straddle the field of view with their full dimensions unavailable, they were excluded from size and shape statistics by excluding all objects with *BorderObject* = 1. Although RAPID is capable of computing several morphological properties (see Supplementary Materials Table S1), only the pore area and circularity were used in this study. All these segmentation parameters can be exported as a *preferences.m* file for future use on a different set of images if necessary. The area, in physical unit lengths, is calculated by multiplying the scaling function K and the total number of pixels of a segmented object (e.g., a pore):

(2) $$A\;=\;K(N_{\mathrm{px}}),$$

where *N_px_* is the number of binary pixels with a value equal to 1. The circularity is calculated as:

(3) $$\mathrm{Circularity}=\frac{4\mathrm{\pi A}}{P^{2}},$$

where *P* is the perimeter (in physical unit lengths). The (field of view) porosity percentage of each specimen was calculated from the following equation:

(4) $$\mathrm{Porosity}\;\%=\left(\frac{A}{A_{\mathrm{FOV}}}\right)\times 100\%,$$

where *A_FOV_* is the area of the entire field of view of the image.

### 2.5. RAPID Tool Validation

The validation of RAPID was performed on several fronts. First, RAPID was validated on use case for porosity characterization (the present study). This validation is meant to provide users of RAPID with an example of how RAPID can be of value. Other example validations that are beyond the scope of this study are available in the Supplementary Materials (Figures S1 and S2). Second, RAPID was validated for intuitiveness in comparison to another established tool (ImageJ/Fiji version 1.54p). This validation is meant to assess the ease-of-use of RAPID and its workflow compared to the other tool. Third, RAPID was validated for its accuracy. The purpose of this validation is to confirm the validity of measurements taken from RAPID against a specimen with known and accurate dimensions.

Given that software intuitiveness cannot be measured directly, a parameter was developed in this study as an indicator for software intuitiveness. This parameter measures the average cognitive load (ACL) the user experiences from the minute they start the software until the segmentation results are saved or exported. ACL was operationalized from the proposed cognitive load theory of Paas et al. and Sweller [18,19,20]. The ACL was calculated as:

(5) $$\mathrm{ACL}=\frac{\sum_{i=1}^{n}C_{i}}{n},$$

where *C_i_* is the interface complexity score for each workflow step, *i* is the specific workflow-step number, and *n* is the total number of workflow steps. The *C_i_* score is measured by directly counting all interactable user-interface elements present on the screen during that workflow step (e.g., menus, sub-menus, buttons, checkboxes, sliders). The theoretical value for ACL is zero, which means the user experiences no cognitive load at any step of the workflow. In other words, if ACL is zero the software handles all workflow steps automatically with no decision or input required from the user. Therefore, the lower the ACL score, the simpler the software is to use. In this study, the ACL scores for RAPID and ImageJ/Fiji were compared for a typical segmentation workflow. This typical segmentation workflow comprised eight steps shown in Table 3.

**Figure 4:**
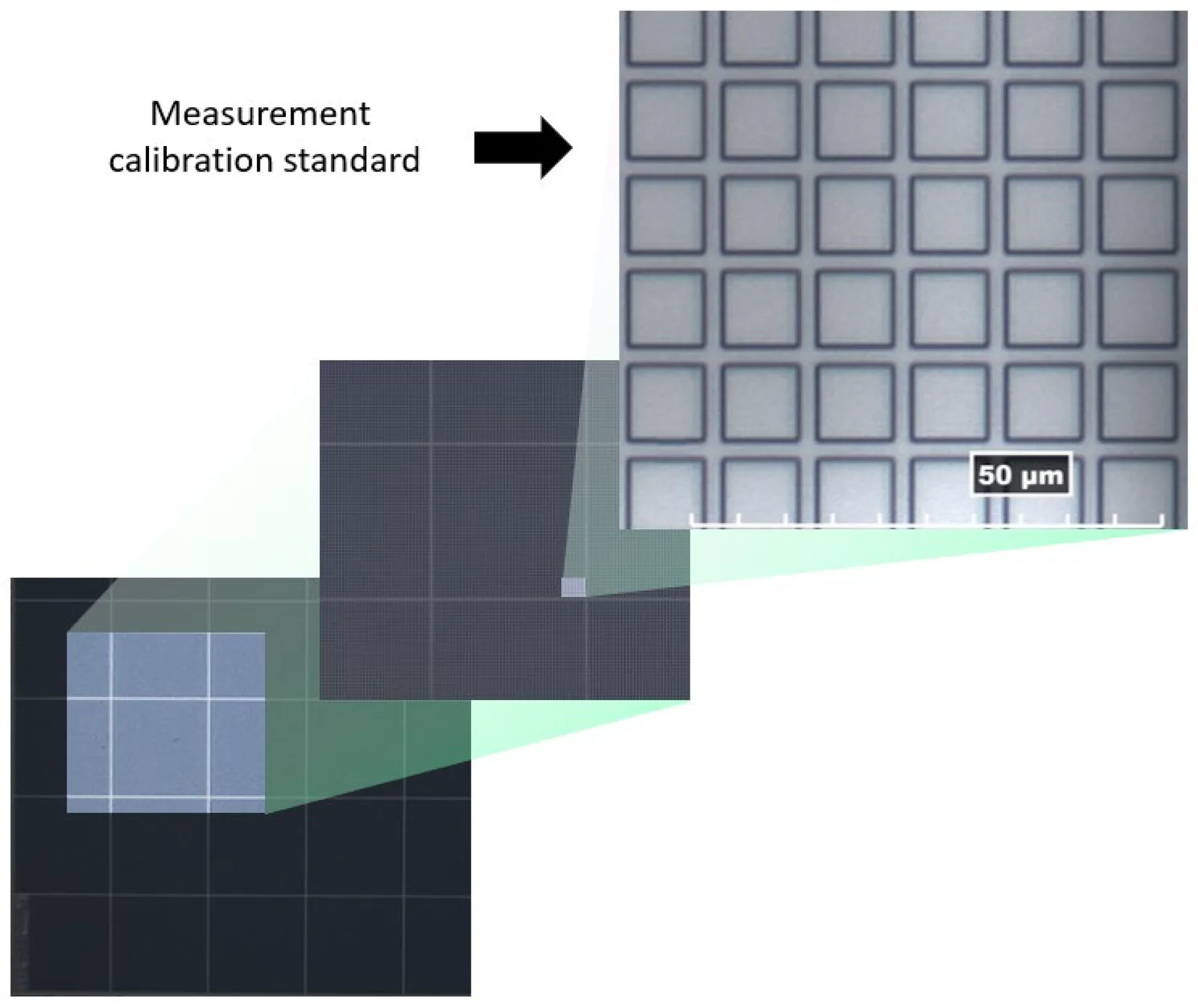
Single-crystal silicon specimen used for measurement validation of RAPID. The nominal pitch within the smallest squares is 10 μm as per the manufacturer.

For accuracy validation, a precision-produced single-crystal silicon specimen with known features was used as a measurement-calibration standard. The specimen was sourced from Agar Scientific (UK). The specimen consists of vertical and horizontal lines etched off a single-crystal silicon substrate, with a nominal pitch within the smallest squares of 10 μm (Figure 4). High-precision etching was performed by the manufacturer using electron-beam machinery. Measuring the pitch can be achieved in several ways, for example, edge-to-edge vertically, edge-to-edge horizontally, centroid-to-centroid vertically, or centroid-to-centroid horizontally. Here, the horizontal centroid method (centroid_x_) was used. The segmentation was performed using the square morphological operator with an edge length of 36 pixels and a binarization threshold of 0.0491. The scale unit was calibrated based on the scale bar supplied with the microscope image, which resulted in 1 pixel = 0.064265 μm. The pitch measurement was performed by taking the distance between the centroids of two adjacent squares along the horizontal axis. The results can be replicated by loading the accompanying data with the manuscript and using the preferences (*preferences.m*) file for pre-processing. After the files are saved, the user can load the saved *mStruct.m* variable to MATLAB^®^. Finally, the user can run the MATLAB^®^ script *AccuracyCheck.m* to obtain identical values.

## 3. Results and Discussion

### 3.1. Characterization of Porosity

A total of 189 surface porosity images were proceed simultaneously using RAPID (methods). The output data from processing these images was obtained in under 8 min on a Windows machine with Intel^®^ Core™ i7-8700K CPU @ 3.70 GHz with 32 GB RAM.

**Figure 5:**
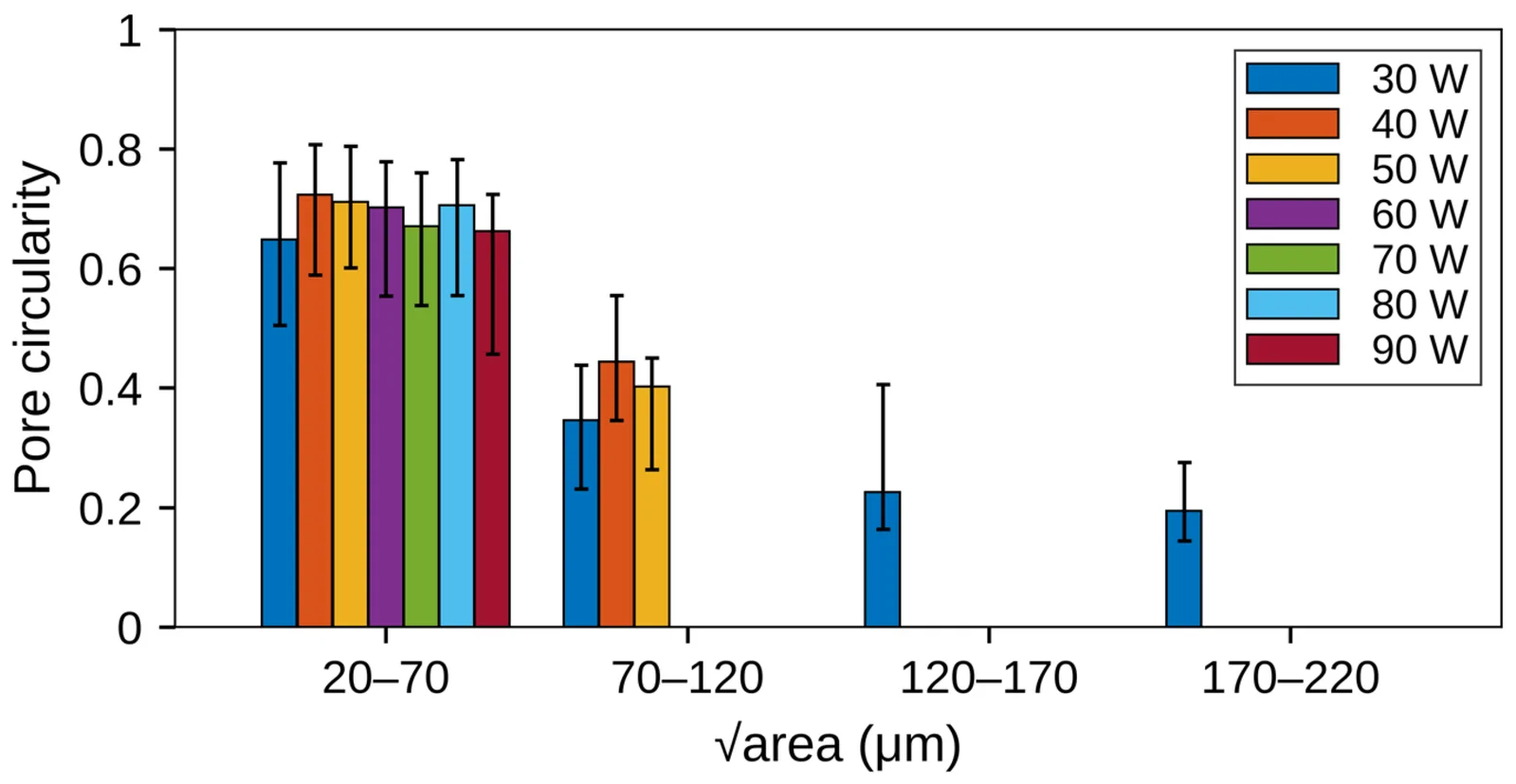
Circularity of pores grouped by size (50 µm). The data reveals that the larger the pores, the more they tend to be irregular in shape (less circular).

**Figure 6:**
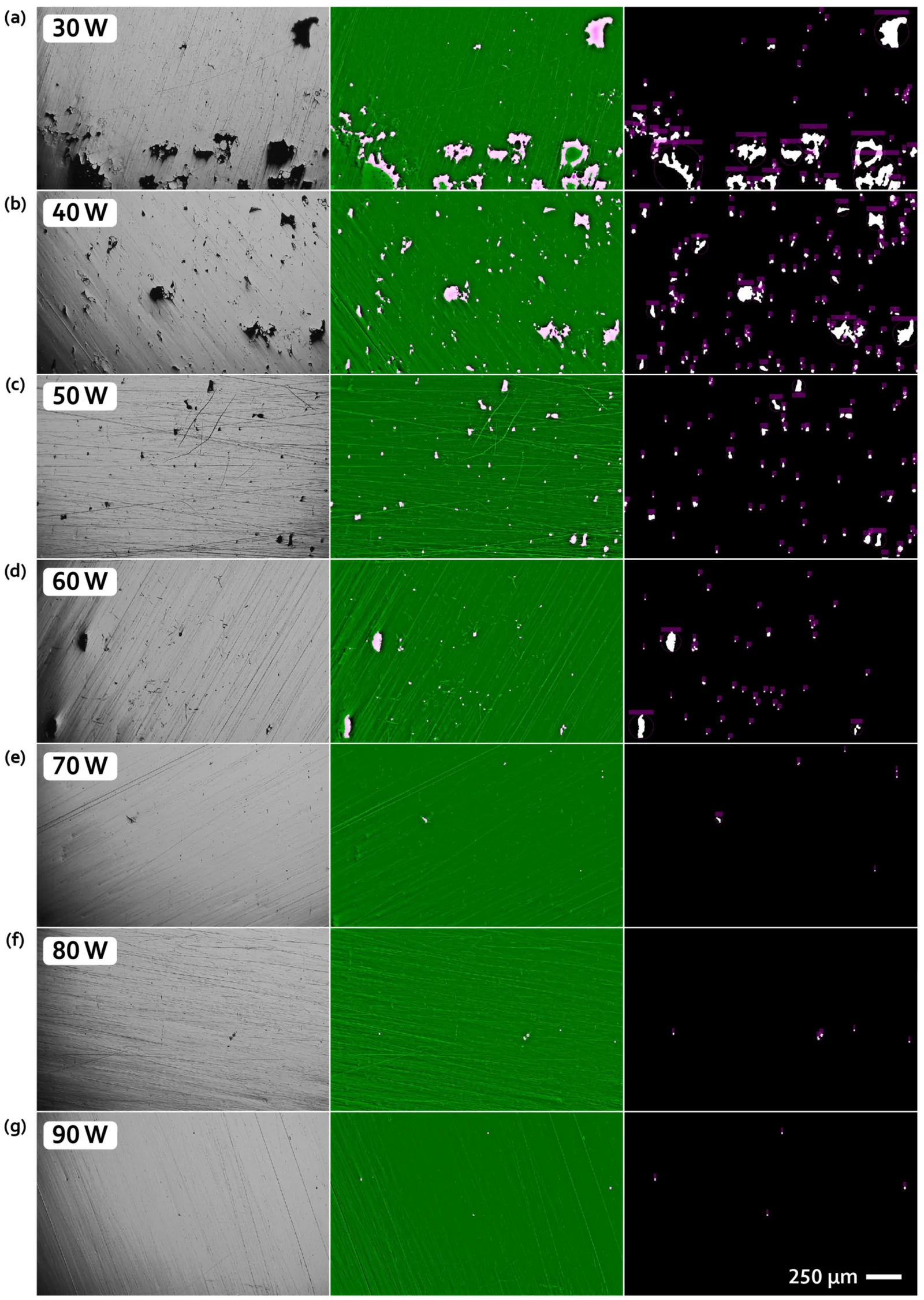
Lack-of-fusion pores per laser power. (**a**) 30 W. (**b**) 40 W. (**c**) 50 W. (**d**) 60 W. (**e**) 70 W. (**f**) 80 W. (**g**) 90 W.

A plot of the pore circularity against pore size is shown in Figure 5. For all laser powers, the data reveals that pore circularity drops as the pore size becomes larger. Most notably, the 30 W specimens (32.5 J/mm^3^) exhibited markedly more irregular pores than the other specimens. These irregular patterns correspond to lack-of-fusion porosities ensuing from insufficient volumetric energy density. Lack-of-fusion porosities were also observed for specimens in the range 30–60 W (32.5–64.9 J/mm^3^) (see representative images in Figure 6b–d). These lack-of-fusion pores seemed to keep on diminishing when the laser power reached ≥70 W (75.8 J/mm^3^) (Figure 6e–g). This observed trend is consistent with Tang et al.’s geometric criterion concerning lack-of-fusion pores [24]. Tang et al. ([24]) stipulated that if the melt pool is sufficiently wide relative to the hatch spacing, and is sufficiently deep relative to the layer thickness, lack-of-fusion pores can be avoided. The geometric criterion is put mathematically as:

(6) $${\left(\frac{h}{W}\right)}^{2}+{\left(\frac{L}{D}\right)}^{2}\leq 1,$$

where *h* is the hatch spacing, *W* is the melt-pool width, *L* is the layer thickness, and D is the melt-pool depth. In this study, *h* and *L* are fixed at 77 µm and 20 µm, respectively, whereas *W* and *D* are governed by the heat flow [25]. Assuming the melt pool is large relative to the thermal diffusion length, Tang et al. proposed an estimate expression for *W* [25,26]:

(7) $$W\approx 2\sqrt{\frac{2Q}{\mathrm{e\pi \rho C}\left(T_{f}-T_{0}\right)V}}\;,$$

where *Q* is the absorbed power (i.e., the product of absorptivity and the laser power), *e* is Euler’s number, *ρ* is the density, *C* is the heat capacity, *T_f_* is the melting temperature, *T*_0_ is the temperature far from the melt pool, and *V* is the scan speed. Given that all parameters in Equation (7) were held constant except for *Q*, it is reasonable to expect that increasing the laser power (and hence *Q*) would in turn increase the melt pool’s width (*W*). Although this assumption may be only partially satisfied at the relatively low laser powers used in this study, the expression nonetheless captures the increase in *W* with *Q*. According to Equation (6), a wider melt pool reduces the first term of the geometric criterion, improving the overlap between adjacent tracks and thereby reducing the susceptibility to lack-of-fusion porosity. This interpretation is consistent with the progressive reduction in lack-of-fusion pores observed with increasing laser power in Figure 6. To the extent that porosity does not vary with depth, the areal porosity results can be used to represent the volumetric porosity (Delesse principle [27]). However, lack-of-fusion pores are notoriously irregular (anisotropic) in geometry. Thus, the measured area of a lack-of-fusion pore regardless of its size can be biased by the sectioning depth. Furthermore, due to the size difference between a lack-of-fusion pore (>100 µm [28,29,30,31,32,33]) and much smaller metallurgical pores (<50 µm [33]), there is a probability bias of encountering the former much more often.

**Figure 7:**
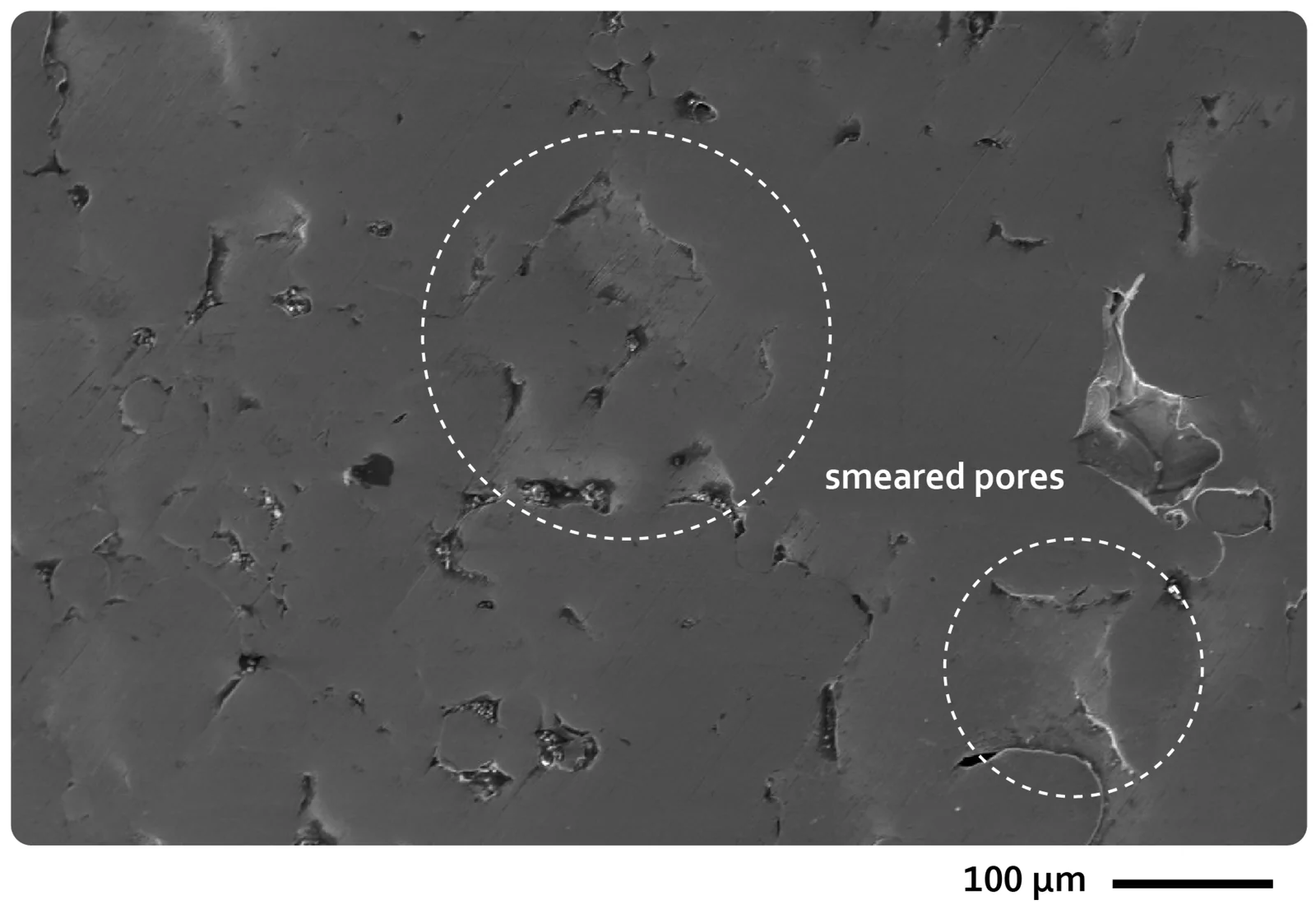
Scanning electron microscopy images showing smeared pores after the polishing of the material.

Post-processing artifacts should also be taken into consideration when measuring porosity via this 2D technique. It can be seen in Figure 6a–g that polishing scratches are present in some samples. These scratches can trigger false detection (as porosities) if the morphological and thresholding parameters in RAPID are not set correctly. If the scratches are too prominent, it may be necessary to pre-process the images by manually removing these scratches to avoid false detection of such features. Furthermore, some artifacts such as smeared pores can arise due to polishing. Pore smearing (or pore filling) can be loosely defined as the transport of material to fill nearby pores [34]. This artifact can give the opposite effect where a pore may be essentially covered by material debris during the polishing process, leading to an underestimate of the porosity. According to Thomas et al., this artifact is especially prominent in softer materials such as copper [35]; however, it is evident from Figure 7 that alloys harder than copper, such as the present 316L stainless steel, are also impacted by this artifact. Since the magnitude of pore smearing was not quantified in the present study, the measured pore sizes may deviate from their true values. However, to mitigate this effect all specimens were prepared using identical procedures (Section 2).

**Figure 8:**
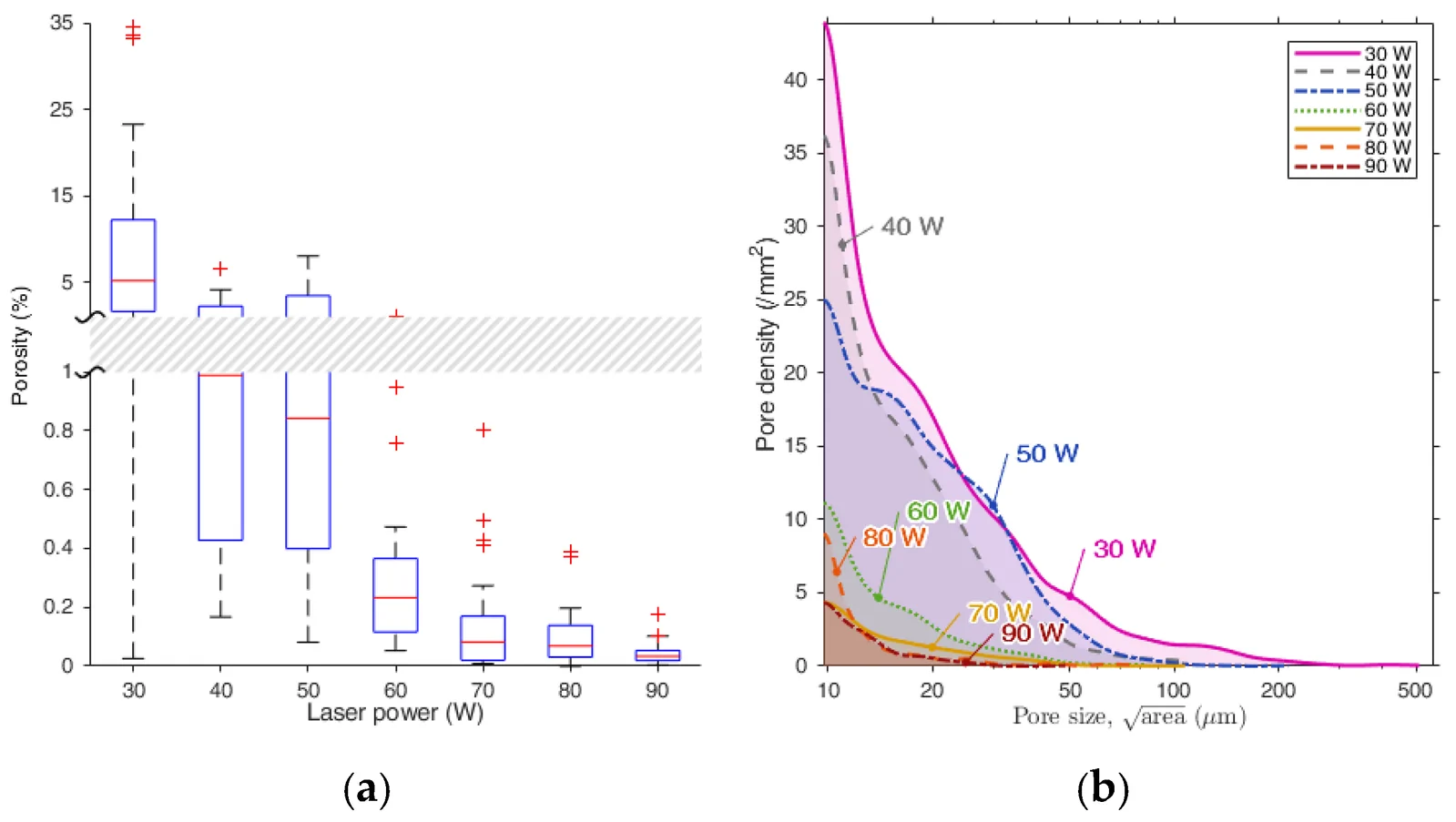
Surface porosity of LPBF 316L stainless steel as a function of laser power. (**a**) Box plots detailing the porosity % for each laser power. (**b**) Pore density (areal density) plotted against the pore size for each laser power.

The box plots in Figure 8a provide a detailed view on the porosity percentages from 27 images per laser power. A clear trend is seen of decreasing porosity percentages with increased laser power; the median porosity percentages consistently decreased from 5.23% (at 30 W) down to 0.03% (at 90 W). Furthermore, laser powers greater than or equal to 70 W exhibited porosity percentages below 1% (including outliers). In addition to porosity percentage, Leuders et al. [36] have shown that the size of pores is also important for the mechanical integrity (i.e., high-cycle fatigue). Figure 8b shows the size distribution of pores and their areal density (pore density per mm^2^) for each laser power. Overall, the areal density and pore size both decrease when the laser power is increased. The largest pore in the 90 W specimen was nearly an order of magnitude less than those found in the 30 W specimens (i.e., 50 µm versus 500 µm). Likewise, the maximum areal density of pores in the 90 W specimens was also nearly ten-fold less than those observed in the 30 W specimens.

**Figure 9:**
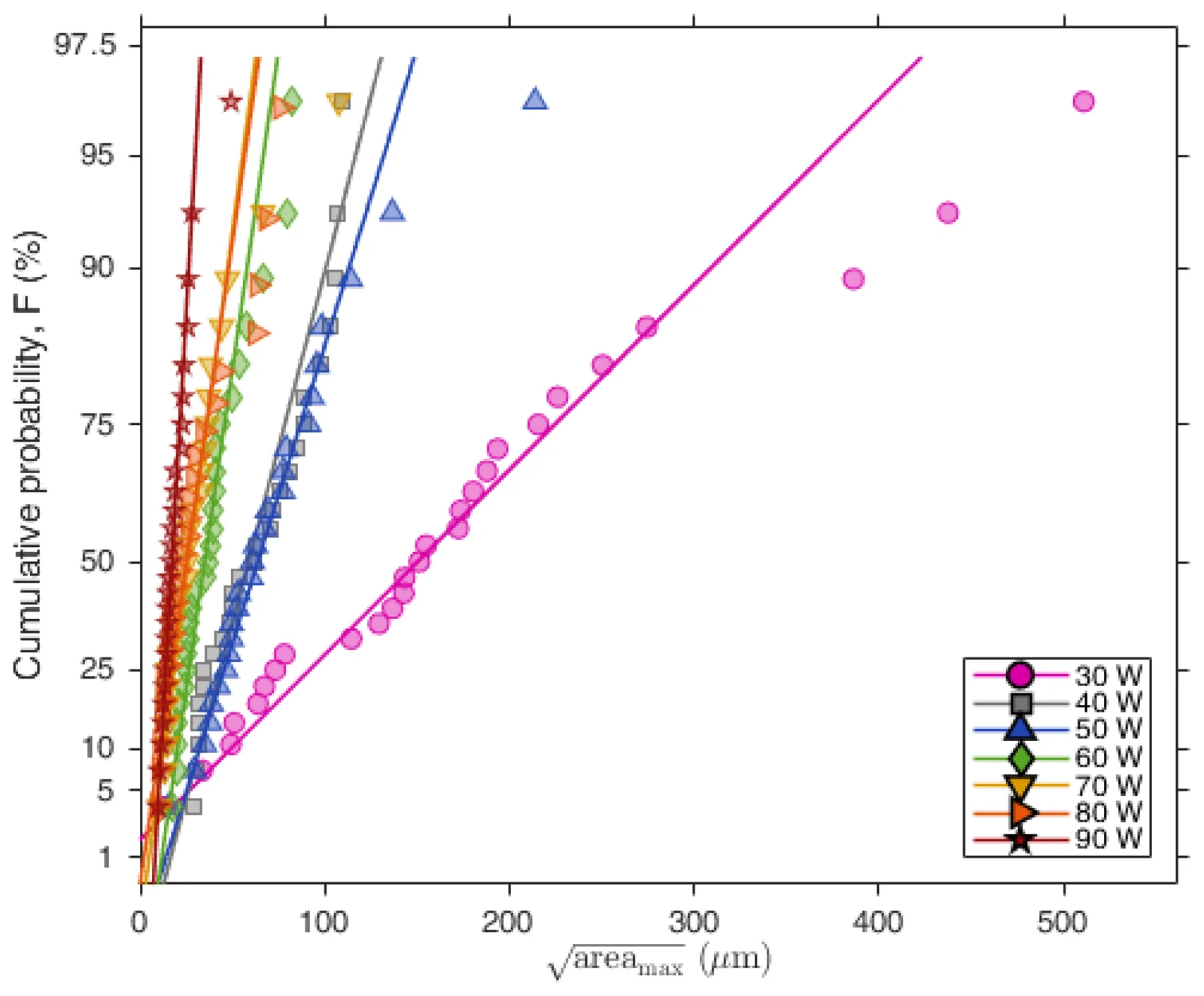
Gumbel plot of largest pore ($\sqrt{\mathrm{Are}a_{\max}}$) in each captured image. The results suggest that the predicted fatigue limit increased 1.5-fold when the laser power was increased from 30 W to 90 W.

A Gumbel generalized extreme value distribution was employed to determine the probability of extremely large porosities existing (which can be linked to fatigue performance) [37,38]. The analysis follows the procedure of ASTM E2283 [39,40], which is the standard practice for extreme value analysis of nonmetallic inclusions in steel and other microstructural features. ASTM E2283 recommends a minimum sample size of 24; the sample size in this study is 27 (images per laser power). The cumulative probability (*F*) for each of these was estimated from its rank:

(8) $$F=\frac{i}{n+1}\;,$$

where *i* is the rank of the pore when the 27 maxima are sorted in ascending order of size and n is equal to 27. As seen in Figure 9, the $\sqrt{\mathrm{Are}a_{\max}}$ values followed their fitted Gumbel line with a few exceptions that fell below the line. The 30 W line exhibited a shallow slope, indicating that its largest surface pores are both much larger and far more variable from image to image than at higher powers. The predicted largest surface pore decreased thirteen-fold from over 390 µm to about 30 µm from 30 W to 90 W, respectively. This decrease corresponds to an approximately 1.5-fold difference in fatigue limit between the 30 W and the 90 W [38]. By comparison, the predicted largest surface pore decreased only two-fold from about 60 µm at 80 W to about 30 µm at 90 W, corresponding to a difference in fatigue limit of about 1.1-fold.

### 3.2. RAPID’s Validation

RAPID was validated in three respects: (1) use-case validation, (2) accuracy validation, and (3) intuitiveness validation.

#### 3.2.1. Validation of Use Case

The use-case validation was on the areal porosity measurement and characterization of LPBF-produced 316L stainless steel imaged with optical microscopy (which is the main theme of this paper). However, RAPID’s use case can be extended to cover other features, materials, and/or imaging techniques. As an example, RAPID can be used to determine the phase fraction of dual-phase stainless steel using optical microscopy (see Supplementary Materials Figure S1). Furthermore, RAPID can be used in tribological analysis to measure the surface wear of a material (see Supplementary Materials Figure S2). Other use cases may be achievable provided that sufficient contrast between the desired object and background is present. However, it is important to note that the optimal parameters used for segmentation directly depend on the features of interest. For instance, the morphological operator used in this study was ‘disk’ because it offers a generic shape suitable for detecting irregular pores. In other applications, a different morphological operator (e.g., ‘square’) can be more appropriate if the features of interest are of square-like shape. The thresholding parameters are also dependent on the illumination and resolution of the images captured, and thus are decided on a case-by-case basis (which can be seen in the example within Supplementary Materials Section S5.1).

#### 3.2.2. Validation of Accuracy

**Figure 10:**
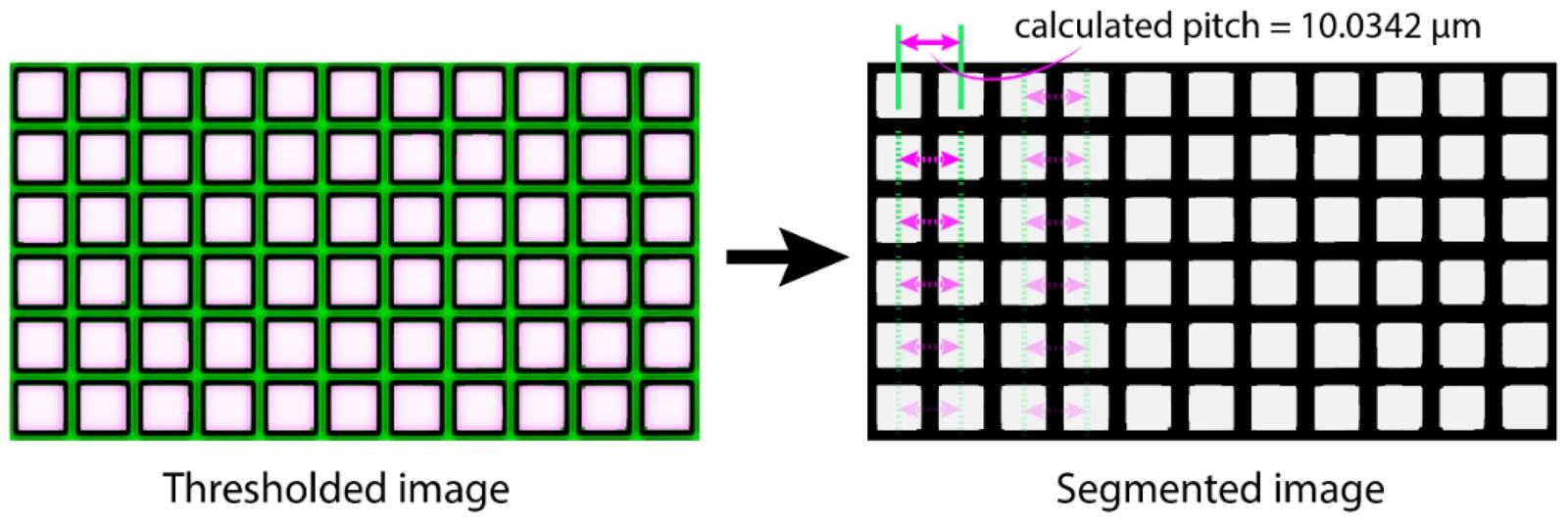
RAPID’s validation against a precision-made, 10 μm pitch specimen. The output result was 10.0342 μm yielding a 99.66% accuracy.

RAPID’s accuracy was validated by measuring a high-precision, 10 μm pitch square array on a silicon specimen. The thresholded and segmented images of the silicon specimen are shown in Figure 10. The mean pitch (*n* = 60) was measured and calculated to be 10.0342 ± 0.0045 μm, which is in strong agreement with the 10 μm reference value (99.66% accuracy). This result validates RAPID’s calibration and use for making measurements.

#### 3.2.3. Validation of Intuitiveness

The intuitiveness of RAPID was validated quantitatively using the average cognitive load (ACL) parameter (see Section 2); To calculate ACL, the interface complexity score (*C_i_*) was first determined. According to Pass et al. [19], a so-called intrinsic cognitive load is imposed by the number of information elements present and their interactivity. This cognitive load interferes with a person’s ability to learn novel tasks. On average, the cognitive load imposed by RAPID is 18.8-times lower than ImageJ’s (Table 4). This procedure was performed having RAPID and ImageJ side-by-side while performing identical segmentation workflow (see Section 2). Furthermore, the *C_i_* for RAPID was consistently and significantly lower than imageJ’s. Notably, this metric does not imply a software’s superiority over the other; rather, it strictly measures the simplicity of using the software.

Although RAPID has been validated for accuracy and intuitiveness, there remain important considerations and limitations. Notably, this study’s results and conclusions involved 2D optical digital microscopy imaging. Despite its utility and cost effectiveness, this technique involves imaging sections of the material’s surface. As such, there is no information regarding the interior porosities or defects. Furthermore, it should be noted that many porosities are anisotropic in nature; thus, the imaged pore can only provide an estimate of the pore’s volume but its reliability is only established based on probability and statistics. The magnification choice (e.g., 20× used in this study) may limit the detection of smaller features (i.e., porosities) as pores smaller than one pixel (e.g., 1.0777 µm in this study) can appear as noise. This limitation can be mitigated by running a second batch with higher magnification (e.g., 50× or 100×) to resolve smaller features [41]. Furthermore, digital imaging can be highly susceptible to artifacts or aberrations caused by improper illumination. Since RAPID uses thresholding techniques, the presence of artifacts or aberrations can lead to false detections. In addition, RAPID works best with grayscale images; any colored images will be automatically converted to grayscale in RAPID. It is necessary to ensure high-quality images (e.g., good focus, no vignetting, uniform illumination) to attain good results with RAPID. RAPID can be used with scanning electron microscopy (SEM) provided that images have clear contrast between the features and background/surface. The same scaling calibration used in the methods can be used for calibrating the measurement with SEM imaging.

## 4. Conclusions

Robust analysis and processing of image data (RAPID) was introduced as a user-friendly, free open-source, and workflow-capable tool for materials scientists and engineers. Here, RAPID was used to study the influence of varying the laser power in steps of ten (from 30 W to 90 W) on porosity count and morphology in 316L stainless steel manufactured by laser-powder-bed fusion technology. All other parameters were held constant. A total of 189 surface porosity images were processed simultaneously at identical parameters. The results showed that:

- Lack-of-fusion pores gradually diminished as laser power was increased to 70 W (75.8 J/mm^3^).
- Increasing the laser power resulted in fewer and smaller porosities. The difference in pore size between the 90 W and the 30 W trials was an order of magnitude (i.e., 50 μm to 500 μm).
- Extreme-value statistics on the largest pore suggested 1.5-fold improvement in fatigue limit when power was increased from 30 W to 90 W, whereas it suggested 1.1-fold improvement when power was increased from 80 W to 90 W.

RAPID’s accuracy was validated against a calibrated standard producing a measurement accuracy of 99.66%. Furthermore, RAPID was validated for average cognitive load (a measure of the average mental effort imposed on a user to complete a task) imposing an 18.8-fold-lower load compared with ImageJ. Ultimately, RAPID is poised as a no-cost open-source, intuitive, and workflow-capable solution that significantly lowers the barrier to entry for quantitative image analysis for material scientists and engineers.

## Figures and Tables

**Table 1:** Nominal composition of the LPBF gas-atomized 316L stainless steel powder.

wt. %												
Fe	Cr	Ni	Mo	C	Mn	P	S	Si	Cu	N	Co	O
Bal.	17.7	12.6	2.38	0.015	0.72	0.009	0.004	0.60	0.02	0.09	ND	0.03

**Table 2:** LPBF process parameters used in this study. All parameters were held constant except the laser power.

Laser Power	VED	Scan Speed	Hatch Spacing	Layer Thickness
30 W	32.5 J/mm^3^	600 mm/s	77 µm	20 µm
40 W	43.3 J/mm^3^			
50 W	54.1 J/mm^3^			
60 W	64.9 J/mm^3^			
70 W	75.8 J/mm^3^			
80 W	86.6 J/mm^3^			
90 W	97.4 J/mm^3^			

**Table 3:** Workflow steps used to calculate the average cognitive load (ACL) for RAPID and ImageJ in the standardized image segmentation process.

Workflow-Step Number (*i*)	Description
1	Start/Load software
2	Load image
3	8-bit conversion
4	Pre-processing
5	Calibrate unit
6	Segment
7	Analyze/Preview segmentation
8	Export/Save data

**Table 4:** The average cognitive load (ACL) comparison between RAPID and imageJ. The *C_i_* values show the interface complexity at each step measured according to Section 2. The ACL imposed by RAPID to the user is 18.8-times lower than ImageJ.

Software	*C*_1_	*C*_2_	*C*_3_	*C*_4_	*C*_5_	*C*_6_	*C*_7_	*C*_8_	ACL
ImageJ	162	162	162	247	172	209	219	171	188
RAPID	2	5	0	22	15	15	7	14	10

## Data Availability

The original contributions presented in this study are included in the article/Supplementary Materials. Further inquiries can be directed to the corresponding author.

## Supplementary Materials

The following supporting information can be downloaded at https://www.mdpi.com/article/10.3390/ma19214527/s1, PDF S1: Core features and functionalities; Video S1: RAPID demo.

## Abbreviations

The following abbreviations are used in this manuscript:

ACL	Average cognitive load
CT	Computed tomography
EED	Enhanced edge detection
GUI	Graphical user interface
IUC	Image uniformity correction
LPBF	Laser-powder-bed fusion
RAPID	Robust analysis and processing of image data
VED	Volumetric energy density
