# Supplementary material for: Fast Characterization of Surface Porosity in Additively Manufactured Components Using the Robust Analysis and Processing of Image Data (RAPID) Tool
Source: Materials (Basel). 2026 Oct 27;19(21):4527. doi: 10.3390/ma19214527 (PMC13763664; doi:10.3390/ma19214527)
Supplement: Supplementary file 1 [file materials-19-04527-s001.zip › PDF S1 core features and functionalities.pdf]

## S5. Supplementary materials

### S5.1. RAPID's other use cases

In this section other use cases of RAPID will be demonstrated apart from the one covered in the main text. The other uses include: 1) Determining phase fraction in duplex stainless steel (DSS), 2) Calculating the area of worn surface in tribological testing.

It is well-known that the ratio of ferrite-to-austinite in DSS results in significant impacts on various properties of the alloy. In this demonstration, it will be shown that a metallurgical researcher can identify this ratio using only RAPID and a digital-microscope image of properly prepared DSS sample. The demonstration is based on the work of Fedorov et al. [1] on annealed DSS at 1200 °C that was subsequently etched with Beraha's agent to reveal the ferrite-austenite phases (Figure S1a). Noteworthy, Fedorov et al. used a microscope equipped with a proprietary image analyzer software from Thioxmet (St.-petersburg, Russia) [2] to determine the volume fraction (Figure S1b). Here, it is shown that such simple analysis can be done without resorting to costly proprietary software.

RAPID was used to segment the DSS image from Fedorov et al.'s work [1]. Replication of the segmentation is possible via the accompanying data in the folder Practical demonstration 1 available on the Github repository. Figure S1c shows the segmented DSS image, highlighting the ferrite phase in black and the austenite phase in white. The total area of the image is 1,344,348 px$^2$ (0.97255 mm$^2$). The sum of the area of all austenite constituents obtained from the data.csv segmentation file was 582,217 px$^2$ (0.42116 mm$^2$). The result is a ferrite volume fraction of 56.69% (compared to 56.96% as per Fedorov et al.'s work [2]). This demonstration shows that RAPID can be used as a no-cost and fast solution to solve simple metallurgical image segmentation problems.

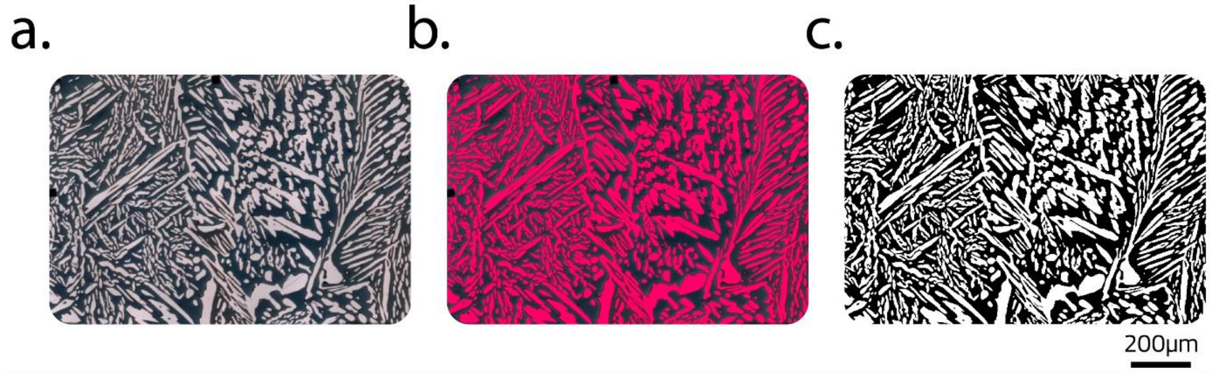


**Figure S1: Phase fraction analysis of duplex stainless steel using RAPID. (a)** Original micrograph of DSS etched with Beraha's agent, **(b)** reference segmentation by Fedorov et al. [1] depicting 56.96% ferrite volume fraction (black), and **(c)** RAPID segmentation depicting 56.69% ferrite volume fraction (in black). **(a,b)** Images from Ref. [1] were obtained under creative commons attribution (CC BY 4.0) license**.**

In tribological testing, it is critical to characterize the wear of the material. There are several methods for measuring and estimating the wear (weight loss, stylus techniques, non-contact optical measurements, etc.). One fast method is to characterize the 2-D surface of the worn volume. RAPID can facilitate the 2-D surface characterization of the worn area. As seen in Figure S2a, the worn area (left) has been detected via rapid using the thresholding technique (right). It is seen in the segmented image (Figure S2b) that several properties are available to assess the morphology of the wear track (Area, major axis, minor axis, circularity, perimeter, etc.). In this instance, the surface area of the wear amounted to 8.29 mm$^2$. Furthermore, it is seen that the major axis and minor axis lengths are 3.94 mm and 2.68 mm, respectively. These properties can be especially useful when comparing the wear tracks of different specimens under variable conditions (load, speed, material, etc.).

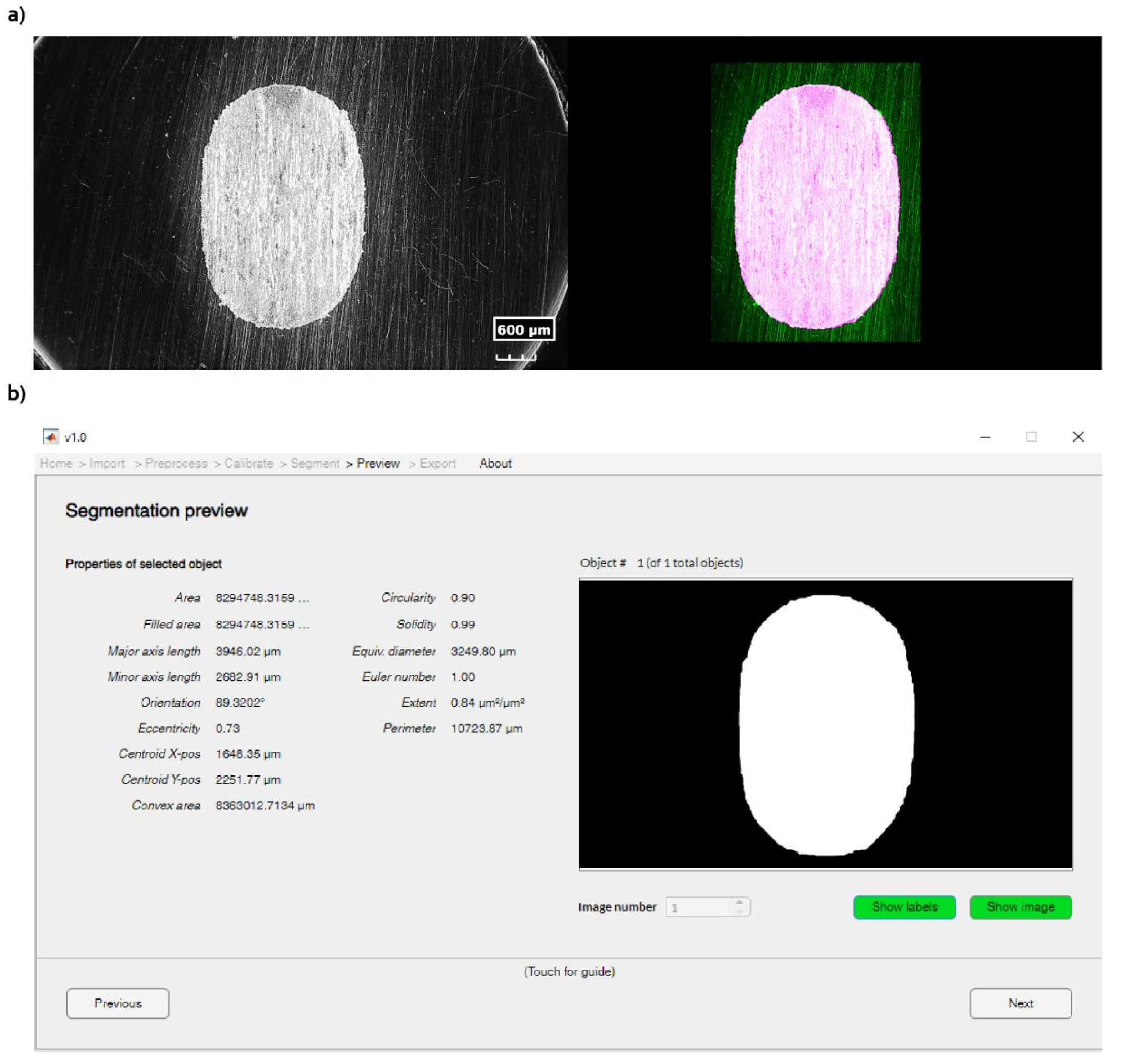


**Figure S2:RAPID's use in measuring the surface wear in tribological testing. (a)** pre-processed image (left) and thresholded image (right), **(b)** segmented image of the wear track and its geometrical properties.

## S5.2. Core features and functionalities

### S5.2.1. Datastore repository

To facilitate efficient use of computer memory, image files are loaded as MATLAB® datastores. Datastores are data management objects that allow the user to access and process large datasets in subsets without loading the entire data into memory.

### S5.2.2. Batch image processing

In many cases researchers need to maintain consistency of image processing and segmentation across multiple sets of images. To facilitate this workflow, RAPID allows the user to load more than one image in a single run (batch image processing).

### S5.2.3. Preprocessing

As already mentioned briefly, preprocessing allows the user to perform certain image processing operations that may aid their segmentation process. Preprocessing can be applied in two methods, manually or uploading a preferences file. The current available preprocessing operations in RAPID are explained below:

- Region of interest (ROI): Enables users to apply segmentation to a selected portion of an image by drawing a rectangle and previewing that region of interest across all image files before confirming (Figure S3a). Utilizing a custom-written script, the user can simply resize the bounding box to the desired region of interest and click on *Crop*. Furthermore, the user can cycle between different images to check whether the drawn region of interest is suitable for all images within the set before confirming the crop.
- Image uniformity correction (IUC): An option to remove vignette artifact (the shading distortion) that often arise from microscope images (Figure S3b). This option utilizes a pre-existing MATLAB® function *imflatfield()*— a function that applies a Gaussian-based, flat-field correction to normalize the intensity variations caused by uneven illumination.
- Enhanced edge detection (EED): A preprocessing feature to enhance the edge contrast to improve object detection in certain circumstances (Figure S3c). A custom function was written to enhance binary edges using successive morphological dilations with linear structuring elements, bridging of small gaps, and hole-filling to improve edge connectivity and object segmentation as detailed below:

The input binary image $I_0$is dilated using linear structuring elements,

$$I_1 = I_0 \oplus (B_{0^\circ} \cup B_{90^\circ}) \tag{S1}$$

Where $\oplus$ denotes the morphological dilation operation, $B_{0^\circ}$ and $B_{90^\circ}$ are the horizontal and vertical structuring elements, respectively. To increase edge continuity, a

morphological bridging operator is applied, followed by a hole-filling operation to recover interior regions,

$$I_2 = \text{fill}(\text{bridge}(I_1)) \tag{S2}$$

Finally, the process of dilation, bridging, and hole-filling is repeated,

$$I_3 = \text{fill}\left(\text{bridge}(I_2 \oplus (B_{0^\circ} \cup B_{90^\circ}))\right) \tag{S3}$$

Where $I_3$ is the final edge-enhanced image.

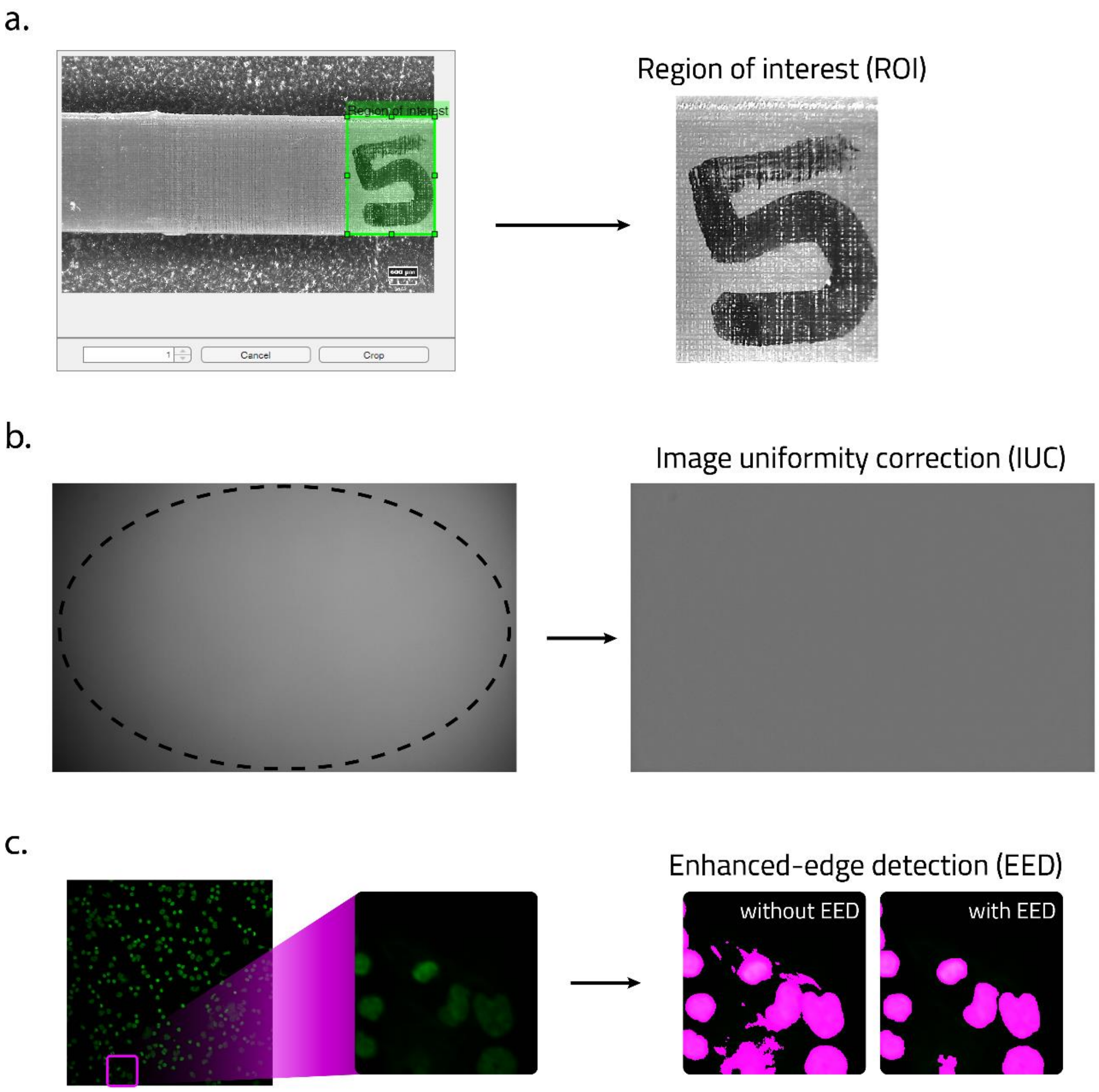


**Figure S3: Preprocessing features in RAPID. (a)** Region of interest (ROI) selection for selective segmentation, **(b)** Image uniformity correction (IUC) for vignette removal using, and **(c)** Enhanced edge detection (EED) for improved object boundary clarity.

### S5.2.4. Preferences file upload

A user can redeploy settings applied to any previous workflow to a new image batch via a preferences file (which is automatically generated after exporting the data). Upon uploading the preferences file, the user is prompted to select which of the options contained in the preferences file they would like to apply to the current set of images (Figure S4). Options include morphological and segmentation operations, pre-processing operations, scaling, and/or export options.

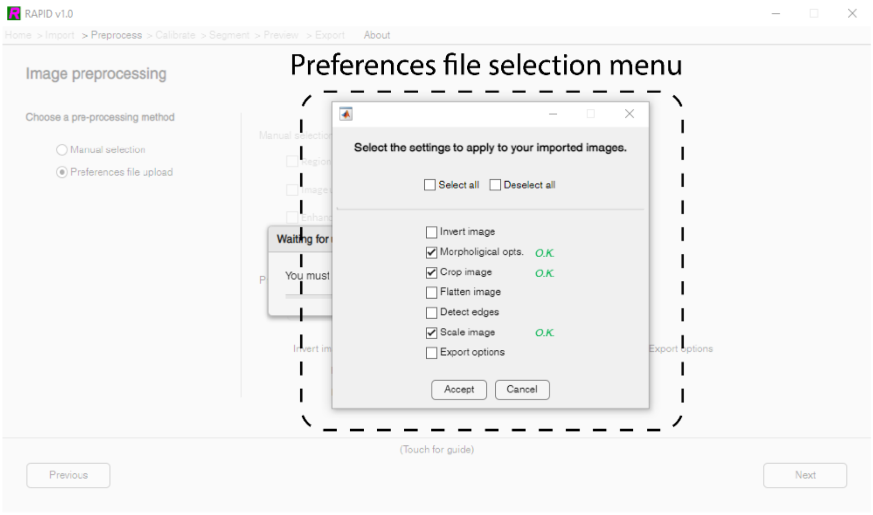


**Figure S4: A selection menu in which user can enable/disable settings from an uploaded preferences file to a new set of images.**

### S5.2.5. Calibration

Instead of using pixels as unit of measurement, a user can calibrate the output unit to a unit of their choice, provided a scalebar with a known length is available. To calibrate, a calibrating image first is uploaded. Then, the user selects from a drop-down menu the unit that matches the unit on the calibrating image (Figure S5, step 1). Then the user enters a number in a textbox that matches the number on the calibrating image (Figure S5, step 1). Next, the user interactively adjusts the length of a visual line to match it against the scale on the calibrating image (Figure S5, step 2). Finally, the user clicks *Confirm* and the unit of measurement is now calibrated (Figure S5, step 3).

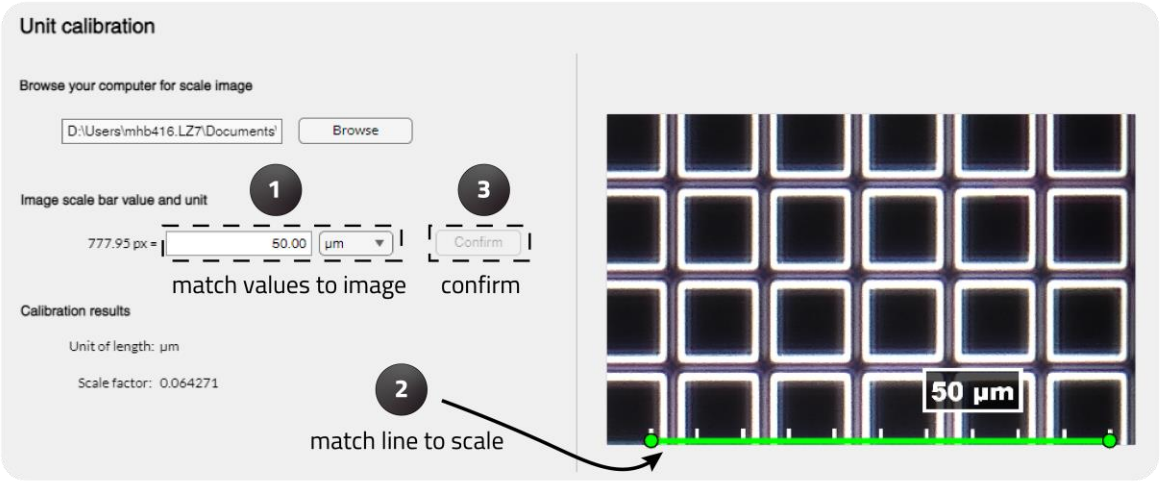


**Figure S5: Calibration interface in RAPID showing the three-step process to scale unit calibration.** (1) Match unit and value with the image, (2) Interactively adjust the line to match the length of the scalebar on image, and finally (3) confirm the changes**.**

### S5.2.6. Morphological shapes selection

To achieve object segmentation, RAPID employs a series of morphological operations and thresholding techniques. Most morphological operations in MATLAB® require defining a structuring element via the *strel()* function [26-28]. This element represents a binary valued neighborhood in 2D whose true pixels are included in the morphological computation, and false pixels are not. Ideally, the shape of the structing element should match the shape of the object which requires segmentation (as to achieve the optimal result). To facilitate this option, RAPID provides the user with the option to select from (Figure S6).

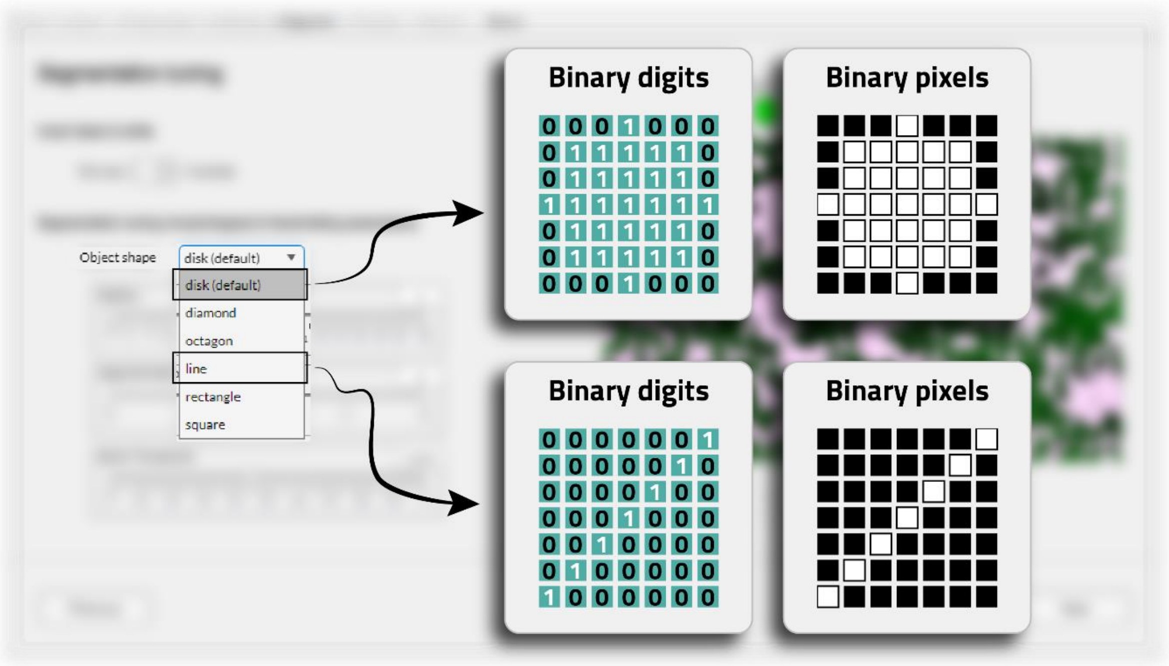


**Figure S6: Morphological shape selection menu in RAPID.** Users can choose a structuring element shape that best matches the object geometry in image for optimal segmentation**.**

### S5.2.7. Real-time segmentation preview

In many situations, the result of a segmentation run can be improved by adjusting the segmentation parameters by means of trial-and-error. However, this approach has the disadvantage of wasting valuable time from researchers. This disadvantage is exacerbated if the size or quantity of images demands high computational power. RAPID overcomes this challenge by allowing the user to visualize the segmentation result in real-time as they fine-tune the parameters (Figure S7).

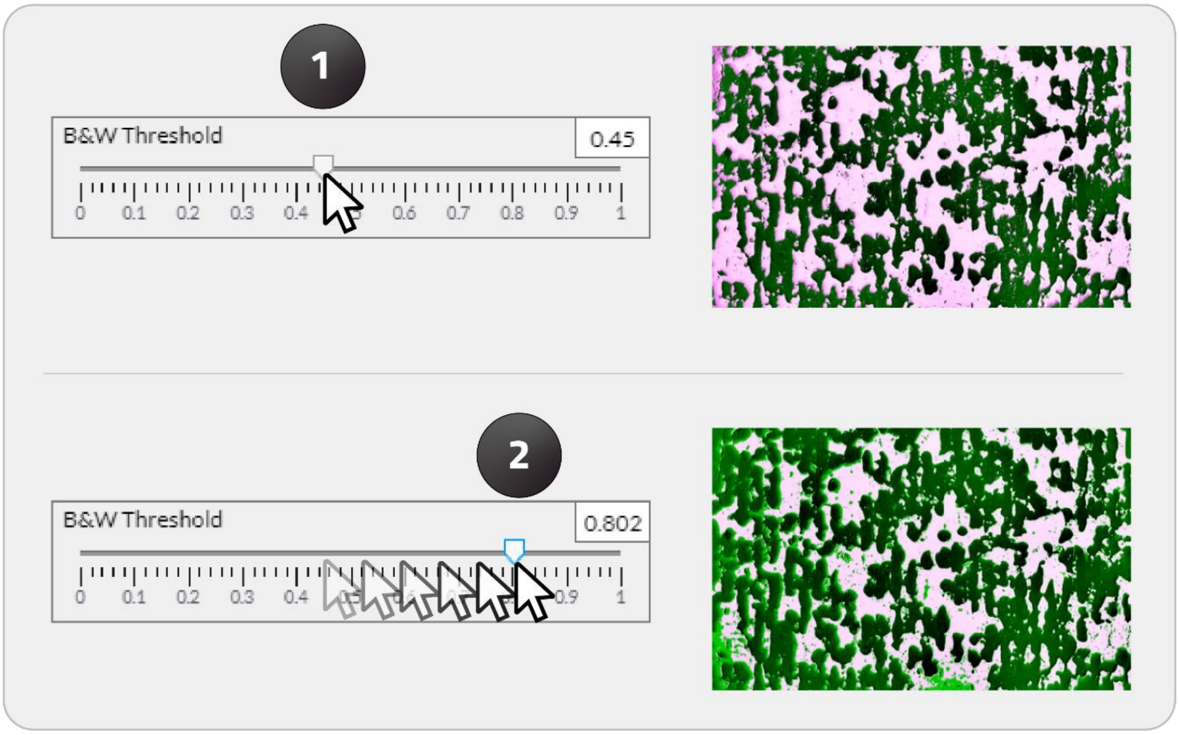


**Figure S7: Real-time segmentation preview in RAPID.** Users can visualize segmentation results to ensure efficient use of resources.

### S5.2.8. Identify border touching objects

Segmented objects can often extend beyond the frame of the image. Including such objects in the analysis may be undesired for some users. Accordingly, RAPID flags such objects with a Boolean operator to give the user the option of in/excluding them in the analysis as needed (Figure S8).

| | A | B | C | D | E | F | G | H | I | J |
|---|---|---|---|---|---|---|---|---|---|---|
| 1 | Unit | FileName | ImageID | ObjectID | BorderObject | Area | FilledArea | MajorAxis | MinorAxis | Eccentrici |
| 32 | px | A1_004.jpg | 1 | 31 | 1 | 48193 | 58289 | 365.7466 | 323.4772 | 0.466673 |
| 33 | px | A1_004.jpg | 1 | 32 | 1 | 1317 | 1342 | 69.37935 | 32.97048 | 0.879867 |
| 34 | px | A1_004.jpg | 1 | 33 | 1 | 23 | 23 | 8.181776 | 4.044795 | 0.869254 |
| 35 | px | A1_004.jpg | 1 | 34 | 1 | 1215 | 1219 | 110.5951 | 34.55762 | 0.949928 |
| 36 | px | A1_004.jpg | 1 | 35 | 1 | 13508 | 14076 | 351.0821 | 85.1279 | 0.970158 |
| 37 | px | A1_004.jpg | 1 | 36 | 1 | 1502 | 1533 | 49.7137 | 42.9724 | 0.502809 |
| 38 | px | A1_004.jpg | 1 | 37 | 1 | 3218 | 3285 | 73.48094 | 62.58635 | 0.523971 |
| 39 | px | A1_004.jpg | 1 | 38 | 1 | 1451 | 1532 | 68.48239 | 36.57576 | 0.845428 |
| 40 | px | A1_004.jpg | 1 | 39 | 1 | 710 | 717 | 66.17765 | 15.1756 | 0.973352 |
| 41 | px | A1_004.jpg | 1 | 40 | 1 | 66 | 66 | 16.02189 | 6.10294 | 0.924611 |
| 42 | px | A1_004.jpg | 1 | 41 | 1 | 23 | 23 | 9.779164 | 3.310438 | 0.940959 |
| 43 | px | A1_004.jpg | 1 | 42 | 0 | 5 | 5 | 2.780887 | 2.780887 | 0 |
| 44 | px | A1_004.jpg | 1 | 43 | 0 | 5 | 5 | 2.780887 | 2.780887 | 0 |
| 45 | px | A1_004.jpg | 1 | 44 | 0 | 99 | 99 | 26.32235 | 6.680695 | 0.967256 |
| 46 | px | A1_004.jpg | 1 | 45 | 0 | 8 | 8 | 3.651484 | 3.05505 | 0.547723 |
| 47 | px | A1_004.jpg | 1 | 46 | 0 | 17 | 17 | 5.671525 | 4.307648 | 0.650482 |
| 48 | px | A1_004.jpg | 1 | 47 | 0 | 22 | 22 | 7.320167 | 4.100898 | 0.828344 |

**Figure S8: Border object detection in RAPID.** Objects extending beyond the image frame are flagged with a Boolean marker (1s are flagged as touching the image frame, 0s are objects that are completely inside the image frame).

### S5.2.9. Interactive preview of segmented objects

RAPID allows the user to interactively click on objects to examine their morphological properties. This feature can be especially useful for examining the morphological properties of outlier data (Figure S9). RAPID gives each object a unique label number (object ID), making it easy for the user to isolate a certain object in the exported tabulated data.

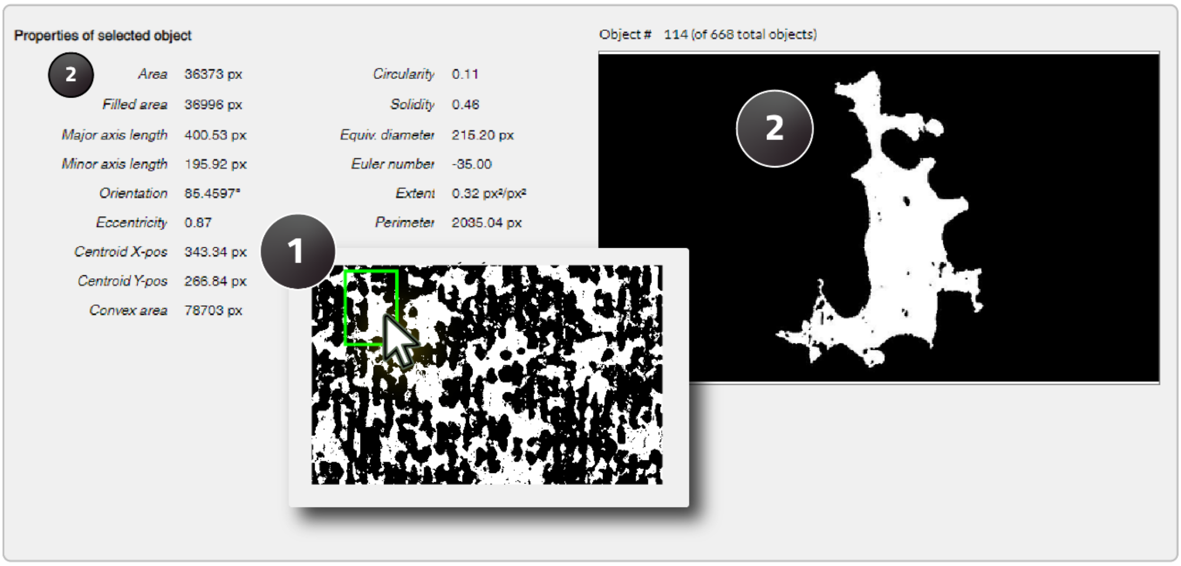


**Figure S9: Interactive object examination in RAPID.** Each segmented object is assigned a unique ID label, allowing users to click and examine an individual object's morphological properties**.**

### S5.2.10. Export options

Users can choose from multiple export options shown in Figure S10. RAPID offers five variations for the exported images. Choosing the *Original images* option lets RAPID include the original (unprocessed) images in the export package (Figure S11a). This option can be helpful if the user needs to replicate or fine-tune the segmentation in the future. The *Binarized images* option is useful if the user needs to have a visual record of the segmentation results (Figure S11b). The *Color-highlighted images* option can be useful if the user wants to perform a visual check on the segmentation accuracy across a large number of image sets. The output of this option is a single image showing the original image on the left against the segmentation visualization image on the right (Figure S11c). The *Labelled-objects images* option is similar to the *Binarized images* option but with the addition of labels on top of the objects (Figure S11d). The option *Binarized images, excluding border-touching objects* will output the binarized images minus all border-touching objects (Figure S11e). Finally, the option *MATLAB® workspace variable* will output a *struct()* variable (mStruct.mat) which can be loaded straight into MATLAB® (Figure S11f). This single variable contains all the segmentation results plus more properties than available through the .csv file (Figure S11g). Furthermore, this option gives the user the flexibility of using MATLAB® as their analysis software if they prefer to do so.

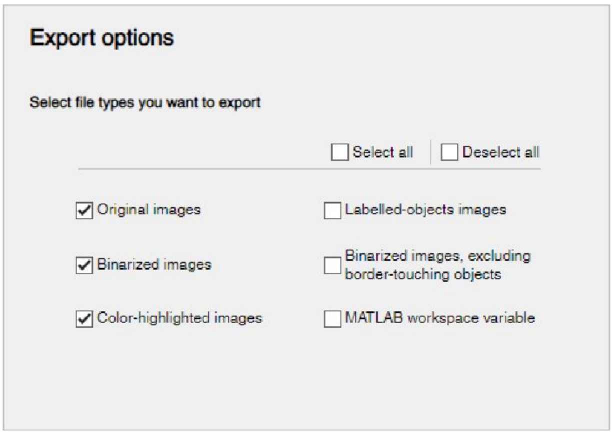


**Figure S10: Export options in RAPID.** Users selections will be included in the saved export folder**.**

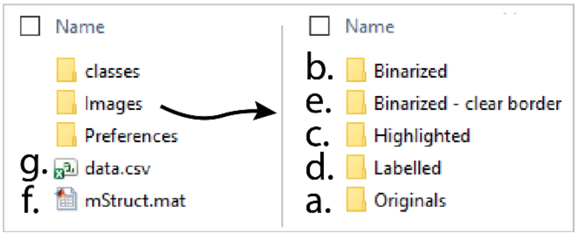


**Figure S11: Export variations in RAPID.** (a) Original images, (b) Binarized images, (c) Color-highlighted images for side-by-side comparison with original images, (d) (binary) Labelled-objects images, (e) Binarized images excluding border-touching objects, (f) MATLAB® workspace variable (mStruct.mat), and (g) comma-separated variable containing the data for analysis**.**

### S5.2.11. Export Table Sproperties

The exported Table S(data.csv) contains many useful properties, some of which are general whereas the others as morphological. These properties are presented and described in Table S1.

**Table S1: Description of exported data properties in RAPID.** The Table Slists general and morphological parameters included in the output file (data.csv). Some descriptions are taken from the *regionprops* page [21] on MathWorks®.

| Property name | Description |
|---|---|
| *Unit* | The unit of measurement. |
| *FileName* | The name of the image file. |
| *ImageID* | A unique ID given to an image when image batch processing is used. |
| *ObjectID* | A unique ID for each segmented object. |
| *BorderObject* | A Boolean to identify objects that are touching the border of the image (i.e., straddle in and out of frame). A value of 0 means the object is completely inside the frame of the image. |
| *Area* | The area of the object in the specified unit of measurement. |
| *FilledArea* | The filled area of the object in the specified unit of measurement. This parameter fills all holes inside the perimeter of the object. |
| *MajorAxis* | The major-axis length of an equivalent ellipse whose normalized second central moments match those of the region, returned as a scalar. |
| *MinorAxis* | The minor-axis length of an equivalent ellipse whose normalized second central moments match those of the region, returned as a scalar. |
| *Eccentricity* | Eccentricity of the ellipse that has the same second moments as the region, returned as a scalar. |
| *Orientation* | Angle between the x-axis and the major axis of the ellipse that has the same second-moments as the region. |
| *ConvexArea* | Number of pixels in *ConvexImage* (the image that specifies the convex hull, with all pixels within the hull filled in (set to on), returned as a binary image). |
| *Circularity* | Roundness of objects. The maximum circularity value is 1. |
| *EulerNumber* | Number of objects in the region minus the number of holes in those objects, returned as a scalar. |
| *EquivDiameter* | Diameter of a circle with the same area as the region, returned as a scalar. |
| *Solidity* | Proportion of the pixels in the convex hull that are also in the region, returned as a scalar. |
| *Extent* | Ratio of pixels in the region to pixels in the total bounding box, returned as a scalar. |
| *Perimeter* | Distance around the boundary of the region returned as a scalar. |
| *Centroid_x* | The horizontal ($x$) coordinate of the center of mass of the region. |
| *Centroid_y* | The vertical ($y$) coordinate of the center of mass of the region. |
| *BoundingBox_x* | The $x$-position of the smallest box containing the region. |
| *BoundingBox_y* | The $y$-position of the smallest box containing the region. |
| *BoundingBox_w* | The width of the smallest box containing the region. |
| *BoundingBox_h* | The height of the smallest box containing the region. |

**References:**

1. Fedorov, A. S., Zhitenev, A. I., Strekalovskaya, D. A., Kur, A. A., Alkhimenko, A. A.: Quantitative description of duplex stainless steels microstructure using selective etching. Metals (2021). https://doi.org/10.3390/met11111750
2. Kazakov, A. A., Kiselev, D.: Industrial application of thixomet image analyzer for quantitative description of steel and alloy's microstructure. Metall. Microstruct. Anal. (2016). https://doi.org/10.1007/s13632-016-0289-6
